# Educational Mechatronics and Internet of Things: A Case Study on Dynamic Systems Using MEIoT Weather Station

**DOI:** 10.3390/s21010181

**Published:** 2020-12-29

**Authors:** Miriam A. Carlos-Mancilla, Luis F. Luque-Vega, Héctor A. Guerrero-Osuna, Gerardo Ornelas-Vargas, Yehoshua Aguilar-Molina, Luis E. González-Jiménez

**Affiliations:** 1 Centro de Investigación, Innovación y Desarrollo Tecnológico CIIDETEC-UVM, Universidad del Valle de México, Tlaquepaque 45601, Mexico; miriam.carlos@uvmnet.edu (M.A.C.-M.); luis.luque@uvmnet.edu (L.F.L.-V.); 2 Unidad Académica de Ingeniería Eléctrica, Universidad Autónoma de Zacatecas, Zacatecas 98000, Mexico; ornelas@uaz.edu.mx; 3 Centro Universitario de los Valles, Universidad de Guadalajara, Ameca 46600, Mexico; yehoshua@valles.udg.mx; 4 Department of Electronics Systems and Computing, ITESO AC, Tlaquepaque 45604, Mexico; luisgonzalez@iteso.mx

**Keywords:** Educational Mechatronics, Internet of things, sensing system, dynamic systems

## Abstract

This paper presents the design and development of an IoT device, called MEIoT weather station, which combines the Educational Mechatronics and IoT to develop the required knowledge and skills for Industry 4.0. MEIoT weather station connects to the internet, measures eight weather variables, and upload the sensed data to the cloud. The MEIoT weather station is the first device working with the IoT architecture of the National Digital Observatory of Intelligent Environments. In addition, an IoT open platform, GUI-MEIoT, serves as a graphic user interface. GUI-MEIoT is used to visualize the real-time data of the weather variables, it also shows the historical data collected, and allows to export them to a csv file. Finally, an OBNiSE architecture application to Engineering Education is presented with a dynamic system case of study that includes the instructional design carried out within the Educational Mechatronics Conceptual Framework (EMCF) to show the relevance of this proposal. This work main contribution to the state of art is the design and integration of the OBNiSE architecture within the EMCF offering the possibility to add more IoT devices for several smart domains such as smart campus, smart cities, smart people and smart industries.

## 1. Introduction

The continuous technological transformation, the industry trends, and the development of new manufacturing processes promote systems’ and processes’ quality and transform machines, work tools, and human beings’ interactions [1]. As part of this transformation, Industry 4.0 (I4.0), has acquired great relevance [2]. I4.0 is characterized by automation, processes’ digitalization, and the use of electronic and information technologies in manufacturing [3]. The Internet of Things (IoT) is a growing technology that has contributed significantly to I4.0, and it allows communication and inter-operation between users and devices that belong to a system [4].

Recently, more companies need to handle the challenge of dealing with the topics of I4.0 and IoT when looking to become I4.0 companies. This is due to the benefits of integrating both topics: traceability, cost reduction, increased productivity, optimized processes and automation, and improved decision making thanks to data collection and analysis. There are several reference models or frameworks devoted to optimize production [5]. For instance, in [6], a service-oriented cyber-physical Advanced Mechatronics System (AMS) was presented along with its current and future challenges. These AMS frameworks show the transformation from mechanical systems to mechatronic systems to advanced mechatronics systems, their interaction with the environment, and other AMSs considering the smart domains and their applications to provide new solutions. Moreover, Reference [7] presented an approach based on the reuse of modular mechatronic systems and a general framework to work with the modular system. It was applied to a six degrees of freedom industrial robot arm and a medical support robot. All the IoT devices and their gathered data could be linked to teaching and learning activities in engineering education as in [8]. The research in the field of IoT applications for smart buildings can be meaningfully interlinked with DigiLab4Ufor a possible laboratory environment to be used in higher education. Furthermore, in [9], the authors presented a framework based on IoT to operate off-line and smart devices; this proposal is divided into three layers: (1) traditional devices layer; (2) the voice and smart device controller are in the same layer to organize the smart devices; and (3) a gateway layer to communicate with the devices. This framework can be applied to smart warehouses, smart restaurants, smart homes, smart farms, and smart factories in which the network may become unstable. It can be noted that those systems in these proposals were adapted or modified, i.e., the complete system was not designed from its conception. This may cause problems when we want to focus on the original system to perform a task or application different from the one for which it was created.

To drive the transformation and ensure success in this new era, training specialists in each area of I4.0 become a key factor. Therefore, it is important to concentrate on engineering education, which is the activity of teaching knowledge and principles for engineering professional practice, not just the methodologies and strategies that are required, but also, a complete educational framework is needed. In [10], an IoT-based learning framework that supports teaching Science, Technology, Engineering, and Mathematics (STEM) was presented. This architecture is devoted to undergraduate education, and it aims to be an environment for teaching computing concepts and theories using a hands-on approach, problem solving, and pedagogy. In terms of hardware and software, they used an Arduino YÚN, ThingSpeak (an open IoT platform with MATLAB analytics), and an Android mobile application. Moreover, in [11], two Industrial IoT devices were developed as a small-scale IoT testbed for both research and education: a 3D printer, vibration data acquisition for machinery health monitoring, intrusion detection in an industrial/manufacturing environment, and an industrial control system emulator. Besides, Reference [12] presented the design and implementation of a new method for the control and monitoring of mechatronic systems, connected to the IoT network using a selected segment of extended reality to create an innovative form of human-machine interface and applying advanced digital technologies such as extended (computer-generated) reality. Although [10,11,12] developed IoT devices and virtual mechatronic systems designed from the ground up, they did not present evidence of any teaching methodology or teaching-learning process. On the other hand, Reference [13] described a framework for the inclusion of IoT in undergraduate curriculum: the outline of the course content, course outcomes, and delivery method. However, this work did not show a conceptual framework containing a methodology, a teaching and learning process, along with strategies to lead the course. It is worthwhile mentioning that one of the novel conceptual frameworks that are just beginning to appear in the academic field is Educational Mechatronics (ME by its initials in Spanish) [14].

Therefore, the motivation of this work lies in the importance of bringing together a complete architecture that considers the new requirements of I4.0 and combines the benefits of IoT applications and an educational methodology, to help institutions, companies government and society to adapt quickly to the exponential growth of technology. Therefore, this proposal encompasses the design and developments of the following elements:

1. An IoT device (called the MEIoT Weather Station) as the first device for the IoT National Digital Observatory of Smart Environments (OBNiSE by its initials in Spanish) architecture, capable of sensing and collecting information about the temperature, humidity, pressure, light, and altimetry sensors.
2. A graphic user interface, GUI-MEIoT, to have access to the data is designed and implemented. The access is available from a mobile device or a computer to monitor and visualize the current data in real-time, make queries, download data in CSV file format, etc.
3. A new IoT architecture, the OBNiSE IoT architecture, that can be applied in general to several areas such as industry, education, mobility, smart cities, agriculture, health, energy, etc. This IoT architecture encompasses six layers: devices, network, processing, cloud, security, and applications.
4. An application to engineering education focused on understanding a dynamic system modeled by a differential equation. Moreover, the instructional design has to be carried out within the Educational Mechatronics Conceptual Framework (EMCF) considering the concrete level, the graphic level, and the abstract level.

The paper is organized as follows. Section 2 presents the definition of the EMCF. Section 3 introduces the OBNiSE IoT architecture, which can be applied to several technological proposals and applications. In Section 4, the detailed description of the design and implementation of the MEIoT Weather Station in the OBNiSE IoT architecture and GUI-MEIoT are presented. Section 5 shows the application to engineering education within the EMCF using the MEIoT Weather Station, and Section 6 discuss the results and how they can be interpreted from different perspectives. Finally, Section 7 draws some conclusions and provides directions for potential future ideas.

## 2. Educational Mechatronics Conceptual Framework

Mechatronics is a multidisciplinary area, as it incorporates elements of electronics, mechanics, robotics, computer systems, control, and manufacturing. Then, there is educational mechatronics, which is developed around this. Educational mechatronics’ main objective is to develop the knowledge, skills, and attitudes required by the new industrial era jobs, promoting students’ critical thinking to generate solutions with innovative proposals to industrial automation problems and processes’ automatic control that industry currently experiences [15].

The Educational Mechatronics Conceptual Framework (EMCF) aims to guide teachers on the design, implementation, and evaluation of pedagogical activities to develop mechatronic thinking in students. The latter is understood as the capacity for the design and implementation of production systems [1] under the principle of interdisciplinary collaboration and the concept of multidisciplinary provision of knowledge [16], in a flexible way [17,18,19], considering the high-level intelligence hierarchy as the backbone of mechatronic systems [15,20]. Educational mechatronics is intended to lead the student to understand abstract concepts on which the applications that we call mechatronics are built and, thus, to be able to face the speed of growth and exponential change of I4.0, the latter with the aim to respond to the manufacturing industry megatrends, focusing on the development, application, or integration of a set of enablers and technologies in order to generate impact [21].

**Figure 1:**
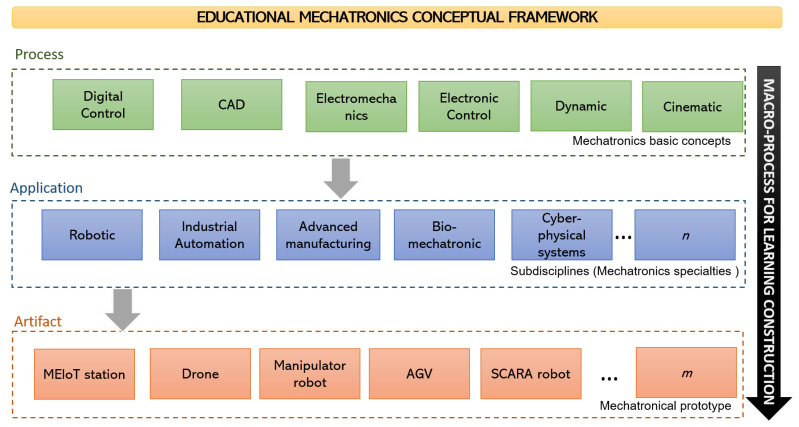
The educational mechatronics conceptual framework and disciplines.

The EMCF is structured into three reference perspectives: process, application, and artifact [22,23], as is shown in Figure 1. The first perspective (process) is oriented toward learning the basic concepts of mechatronics applied to any process of the EMCF, such as the concepts of digital control, electronics, dynamics, and cinematics, among others. The second perspective (application) comprehends all the applications (sub-disciplines) from the basic definitions of mechatronics, which means that students use applications required to present results and be aware of the concepts that are applied during the application use. Some of the applications that students can use are: robotics, cyber-physical systems, biomechatronics, etc. Finally, the last perspective (artifact) is oriented toward getting some models and prototypes related to the process and application construction; these models or prototypes can be a drone, the MEIoT Weather Station, a manipulator robot, or others.

**Figure 2:**
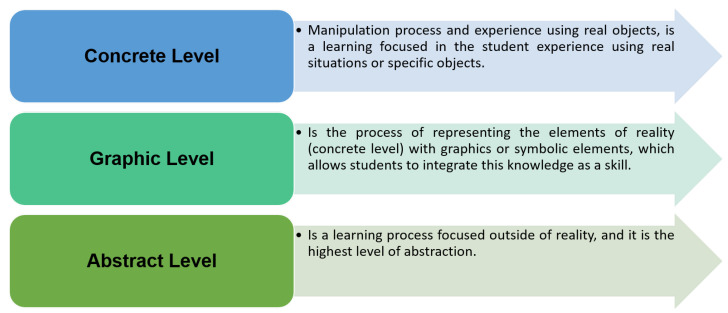
Educational mechatronics conceptual framework macro-process levels and processes involved in the construction of learning.

The EMCF learning construction methodology (macro-process) is based in the structured teaching methodology proposed by [24,25], shown in Figure 2.

This methodology is composed of three learning levels: concrete, graphic, and abstract learning. The first level, called concrete, is about the manipulation process and the experience with real objects, which is a learning framework focused on the student’s experience with situations of their reality or specific objects [26,27]. At the second level, graphic, the elements of reality (defined in the concrete level) are represented using graphics or symbolic elements, enabling students to integrate this knowledge as a skill [28]; the student is able to use applications available online or create new ones. The third level, abstract, is about the learning-focused outside of reality and is responsible for a major abstraction level; at this level, students differentiate between the acquired mathematical and computational knowledge and how this is used within the application.

## 3. OBNiSE Architecture

The Internet of Things (IoT) is one of the most popular technological development areas today due to the demand for current technology and the ease of development it offers. The new methodologies, methods, and architectures have to be capable enough so that they can process the large amount of information generated by different devices connected to the cloud and, at the same time, to ensure users’ and applications’ data.

**Figure 3:**
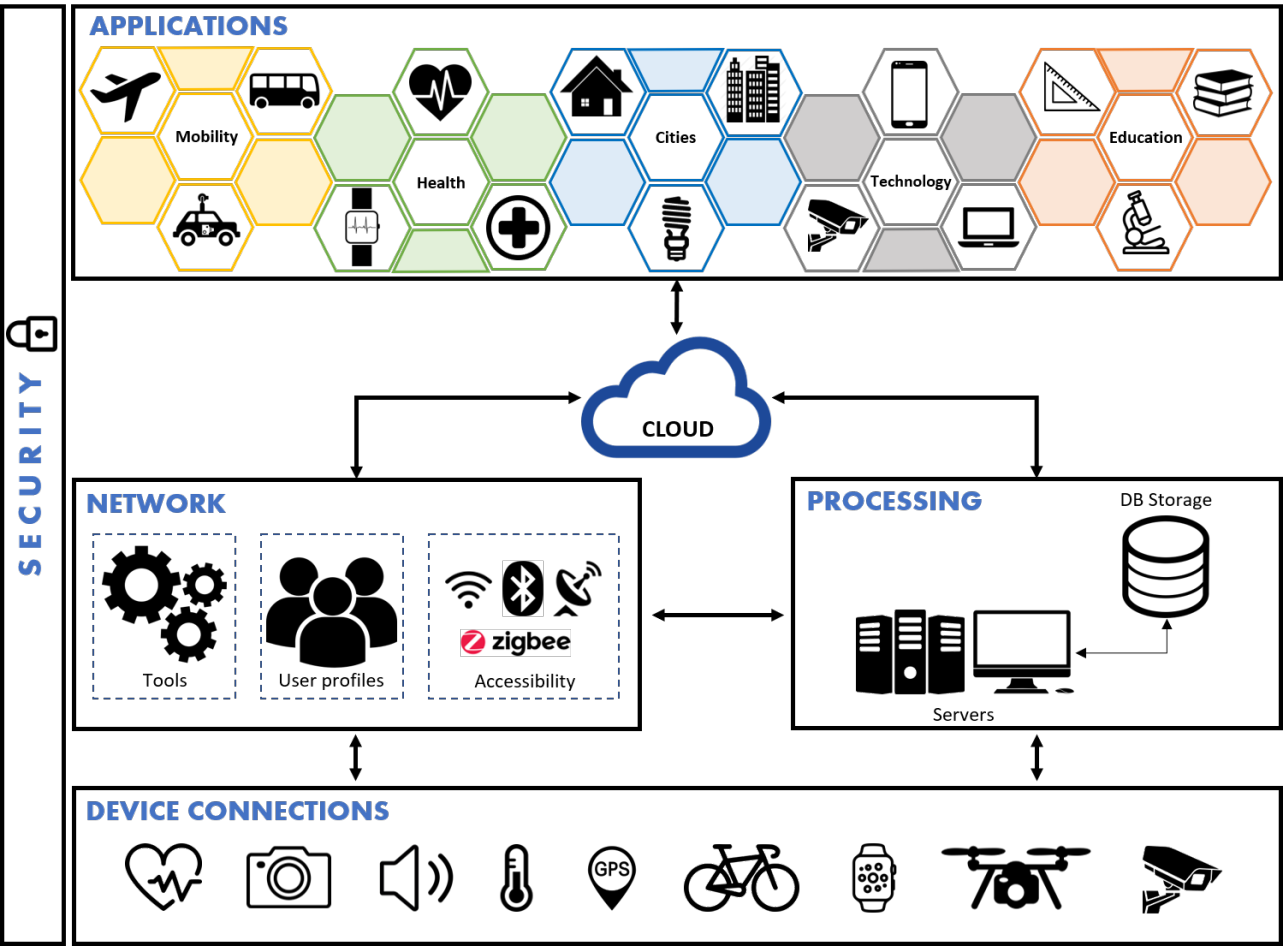
Internet of Things OBNiSE architecture.

This work uses OBNiSE and proposes the OBNiSE architecture: a scalable and multifunctional platform that allows the integration of artificial intelligence mechanisms, distributed systems, networks, and information technologies, among others, in order to obtain new technologies based on IoT (see Figure 3), which has the objective of being an integrated system that allows us to control, analyze, monitor, and develop intelligent systems for the creation of IoT-based solutions, and the security and handling of large amounts of information at different levels are considered. This architecture is distributed in different layers that allow managing and ensuring the availability and security of users, applications, and systems. It is worth mentioning that the OBNiSE is part of the Center for Research, Innovation, and Technological Development of the University of the Valley of Mexico (CIIDETEC-UVM). One of the works that has been developed in OBNiSE is the implementation of an intelligent parking sensor shown in [29].

The OBNiSE architecture is divided into six layers.

1. Devices are all the devices in the physical layer that allow the collection of information.
2. The network is responsible for managing three main components: tools, user profiles, and data accessibility. Tools are the cables, graphics cards, cards, and additional connections that allow users and devices to be configured. User profiles can be divided into three categories: programmers, managers or admin, and regular users. Data accessibility is how devices communicate with users and other devices, whether through Wi-Fi, Bluetooth, and ZigBee, among others.
3. Processing is in charge of processing the information captured by the network and device layers; additionally, it is in charge of data organization to obtain the data interpretation. This processing can be done through a physical or virtual server.
4. The cloud is this layer where the information is stored and ensures its availability for any user or device that uses any application or service of this architecture.
5. Applications allow users to interact with applications and services created under this IoT architecture that offer a technological solution; some of the areas for these solutions are education, mobility, cities, health, and technology, among others.
6. Security is the layer transversal to all the architecture layers. In the case of the devices, the connections are ensured, and the devices are stored in controlled places, while in the user profiles (network layer), it is ensured that they can only see the information to which they have access. The applications contain encrypted information that allows them a level of security such that the information is stored on a physical server and servers in the cloud so that only administrators have access to it. In the processing layer, the server contains encryption keys to protect the information that is transmitted to the devices and the cloud.

## 4. Design and Implementation of the MEIoT Weather Station in the OBNiSE Architecture

**Figure 4:**
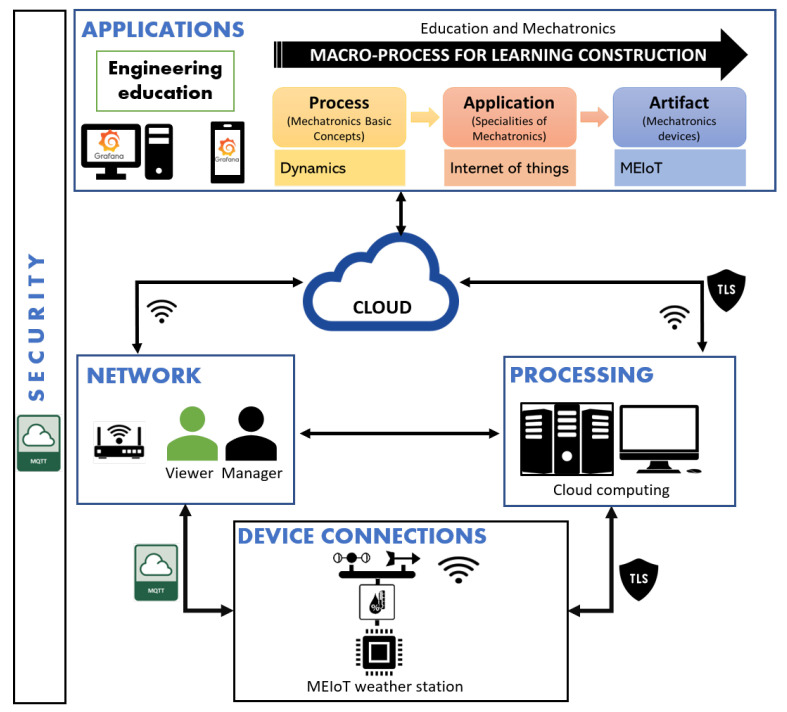
MEIoT Weather Station based on the OBNiSE architecture.

The MEIoT Weather Station was chosen to be the first IoT device in the OBNiSE architecture since it is composed of temperature, relative humidity, barometric pressure, altitude, light, rainfall, wind speed, and wind direction sensors. The OBNiSE architecture considering the MEIoT Weather Station is shown in Figure 4. Moreover, the description for every layer in the OBNiSE architecture is described below.

**Figure 5:**
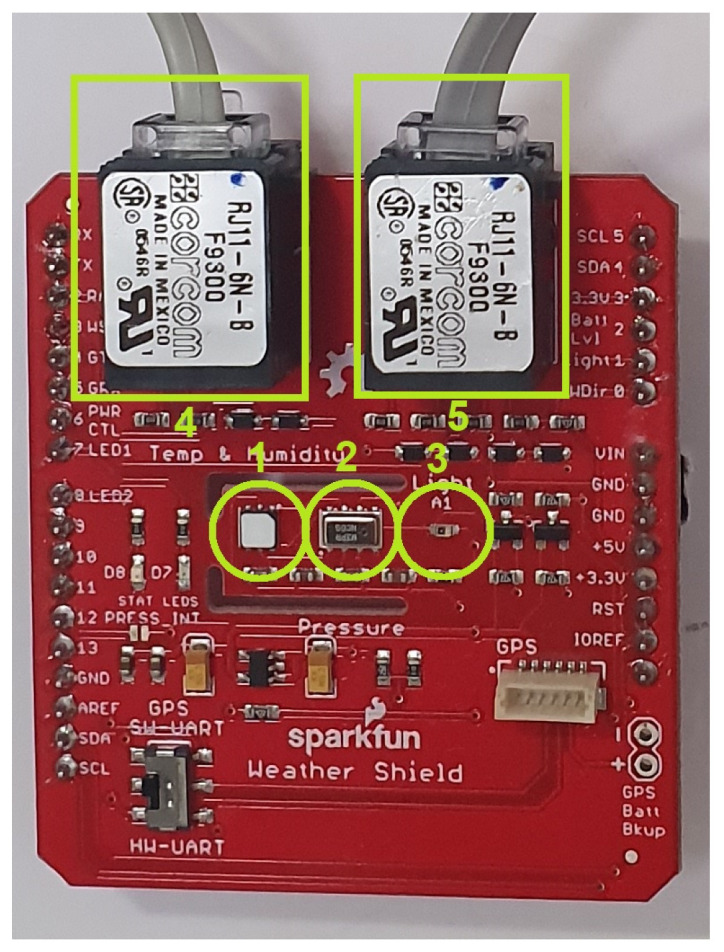
MEIoT Weather Station sensors.

**Figure 6:**
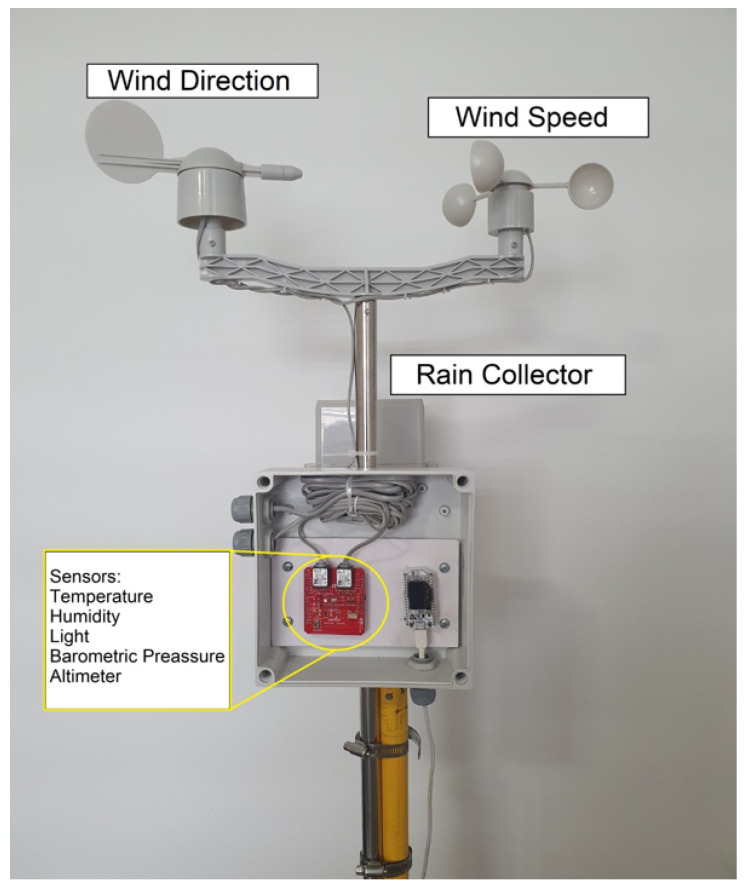
Installed MEIoT Weather Station.

- Device layer: The MEIoT Weather Station uses an Arduino-based microcontroller, temperature, humidity, light, barometric pressure, and altimeter sensors, a rain collector, and wind direction and wind speed elements, as shown in the Figure 5.
  A Si7021 I2C Humidity and Temperature Sensor from Silicon Labs (Circle Number 1 in Figure 5) is a monolithic CMOS IC (Complementary Metal Oxide Semiconductor Integrated Circuit) that integrates humidity and temperature sensor elements, an analog-to-digital converter, signal processing, calibration data, and an I2C (Inter-Integrated Circuit) interface. The patented use of industry-standard, low-Kpolymeric dielectrics for sensing humidity enables the construction of low-power, monolithic CMOS sensor ICs with low drift and hysteresis, and excellent long-term stability.
  The humidity and temperature sensors are factory-calibrated, and the calibration data are stored in the on-chip non-volatile memory. That ensures the sensors are fully interchangeable, with no recalibration or software changes required, and it features a relative humidity precision of ±3 percent maximum in the zero to 80 percent range and a ±0.4 °C maximum in the −10 to 85 °C range.
  The I2C Precision Altimeter MPL3115A2 (Circle Number 2 in Figure 5) employs an MEMS (Microelectromechanical Systems) pressure sensor with an I2C interface to provide accurate pressure/altitude and temperature data. The sensor outputs are digitized by a high-resolution 24 bit Analog-to-digital Converter (ADC). Internal processing removes compensation tasks from the host MCU (Microcontroller Unit) system. Pressure output can be solved with output in fractions of a Pascal, and altitude can be resolved in fractions of a meter. The package is a surface-mounted device with a stainless-steel lid and is RoHS (Restriction of Hazardous Substances) compliant.
  The ALS-PT19-315C/L177/TR8 (Circle Number 3 in Figure 5) is a low-cost ambient light sensor, consisting of a phototransistor in a miniature SMD (Surface-Mounted Device). The spectral response of the ambient light sensor is close to that of human eyes, with a light to current analog output and good linearity across a wide illumination range, which makes it ideal for the MEIoT Weather Station.
  The MEIoT Weather Station also includes a wind vane, a cup anemometer, and a tipping bucket rain gauge; see Figure 6 for more details; Circles 4 and 5 in Figure 5 refer to the connections to the MEIoT Weather Station board. These sensors contain no active electronics; instead, they use sealed magnetic reed switches and magnets to take measurements. A voltage must be supplied to each instrument to produce an output. The rain gauge is a self-emptying tipping bucket type. Each 0.2794mm of rain causes one momentary contact closure that can be recorded with a digital counter or microcontroller interrupt input.
  The cup-type anemometer measures wind speed by closing a contact as a magnet moves past a switch. A wind speed of 2.4 km/h causes the switch to close once per second. The wind vane is the most complicated of these three sensors. It has eight switches, each connected to a different resistor. The vane’s magnet may close two switches at once, allowing up to 16 different positions to be indicated. An external resistor is used to form a voltage divider, producing a voltage output that can be measured with an analog to digital converter.
- Network layer: The sensor and the microcontroller are connected using the I2C (Integrated to Circuit) communication protocol bus, analog ports, and external interrupts, and VNC (Virtual Network Computing) is used for the communication between the CPU (Central Processing Unit) and the mobile application through the terminal. Finally, the information is sent to the cloud using Wi-Fi. The network layer is integrated by three elements: tools, user profiles, and data accessibility. Each item is composed as follows:
  − Tools: In addition to the tools required to connect the sensors and the MEMS using an I2C integrated and VNC, cables, connectors, and additional tools are also part of this element.
  − User profiles: For these implementations, two kinds of user-profiles are defined: viewers and managers; viewers can only see the information using a PC or a mobile; managers, on the other hand, not only are able to see the information, but also have the possibility of modifying the parameters within the MEIoT Weather Station. These profiles are explained in Section 4.2.
  − Data accessibility: This element is used to define the communication channel for the devices, users, and information. For this implementation, the protocols used are Wi-Fi and the MQTT (Message Queuing Telemetry Transport) protocol.
- Processing layer: The MEIoT Weather Station uses a C++-based system with the ability to connect to the Internet and sense and store the data for subsequent information management. The information is stored using cloud computing. The device contains an ESP32 processor, which has built-in Wi-Fi capability; this is used to send the sensors data to the IoT services offered by IBM’s cloud. IBM has the Watson IoT platform, which is its specialized platform for IoT.
- Cloud layer: The information is stored using a database in the cloud for later visualization. The data are available for the application and the system. Due to the lack of access to the OBNiSE facilities due the current pandemic, the IBM Watson platform is currently used for information storage.
- Applications layer: For data visualization, an open-source platform named Grafana Cloud is used, which allows interaction with the MEIoT Weather Station from a mobile device, a computer, or the web. More details are explained in Section 4.2.
- Security Layer: Security is considered in connected devices, devices, and users. It is necessary to define modifications in the configurations for the data protection, the use of the application at the user profile and application levels, as well as the processing and cloud data storage. The security implementation starts with the Watson IoT platform where unique organization IDs are assigned. The IBM Watson IoT platform has three main aspects of security, Transport Layer Security (TLS), authentication, and authorization. (1) The TLS certificate protects against impersonation by certifying a public key belonging to a specified entity. TLS provides secure communication for messages flowing between the client and the IBM Watson IoT platform and supports multiple versions; TLSv1, TLSv1.1, TLSv1.2. (2) The authentication uses a token that is defined in the platform; if any user, device, or connection is successfully authenticated, then, it can be connected to the platform. (3) The authorization is based on a mechanism to allow clients to connect and use messaging actions. There are two types of policies: connection and messaging policies. Connection and messaging policies provide access control. Access control defines which clients are allowed to connect to the Watson IoT platform. Access control also defines the actions that the connected clients are authorized to use. The client identification data must contain the client ID and user ID. The device must present these credentials in order to send data to the organization, and this connection is achieved through the MQTT protocol.

More details about the device connection can be found in the next subsection. An adaptable model for the architecture based on IoT is presented. This model is configurable according to the needs of data growth and current technological advancement that requires tools that are sufficient and competitive in this new industrial revolution.

### 4.1. Description of the MEIoT Weather Station

**Figure 7:**
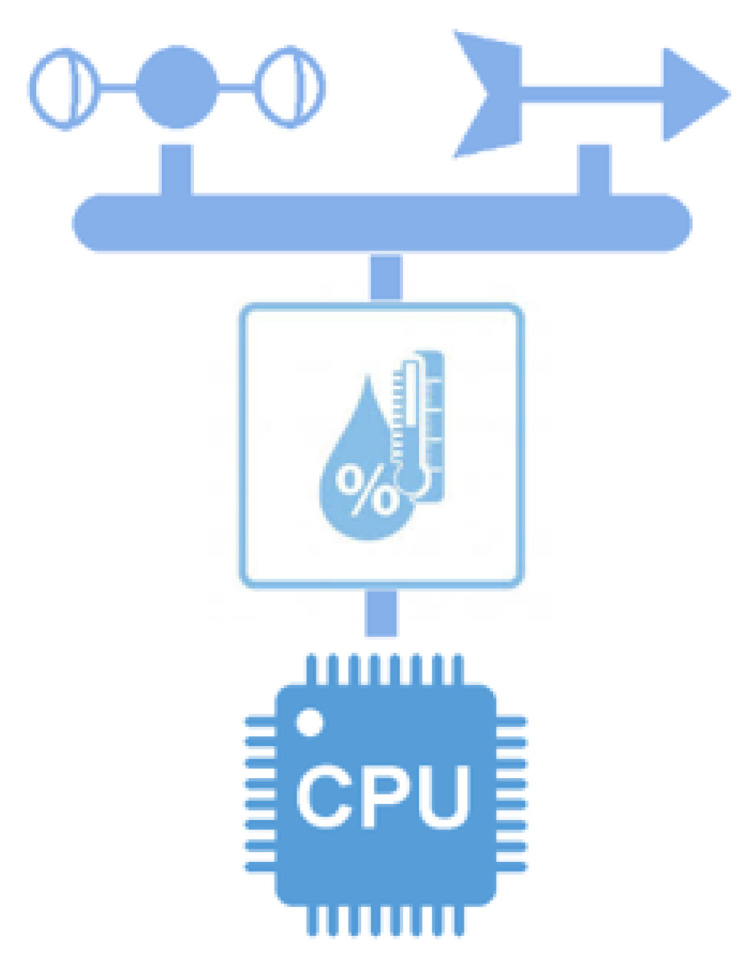
MEIoT Weather Station icon.

The MEIoT Weather Station is a facility with instruments and equipment for measuring atmospheric conditions to provide information for weather forecasts. This information can be used for different purposes. The MEIoT Weather Station is composed of an Arduino-based microcontroller that contains a Control Processing Unit (CPU) to process the information. The CPU is connected to the sensors to receive the sensed data. The CPU is also connected to the cloud to store the sensed data via Wi-Fi. Figure 7 depicts the icon used for the MEIoT Weather Station.

The multidisciplinary nature of this system requires three fundamental phases for its design and implementation process: cloud-based database design, communication of elements, and open-source website platform.

There are two options when designing the database: the relational model and the entity-relationship model. In this work, the relational model was developed for this system. In order to organize and easily access the data, a database is in charge of storing the measured sensors data, the sampling time, as well as the date of the sample; they can be consulted by the website platform.

Using an Arduino-based microcontroller board as a CPU, sensors are connected using the I2C communication bus, analog ports, and external interruptions. Starting from this, an embedded system is developed in the C++ language to connect to the Internet and capture the sensors’ obtained data. The same program has the task of establishing a connection with an online database and sending the measured data with a sampling time of 1 s.

The physical interconnection between all elements is shown in Figure 6, where a printed circuit board (red board) interconnects the weather sensors; this board is also connected to the microcontroller board (white board). The electronics are arranged in a small plastic enclosure mounted on a tripod for practical usage and can be moved to a specific location for running tests in different environments.

### 4.2. Graphic User Interface-MEIoT

Several applications can be used for the visualization and manipulation of the data of an IoT application. For the online platform, we use Grafana Cloud, an open-source platform for querying, visualizing, and alerting metrics and logs, wherever they live. The navigation of the website is intuitive and easy for the user to use. The platform also allows setting the framing and the start and end time to show the later graphics. The user also can download the proportions from the website in a CSV (Comma-Separated Value) format.

Grafana offers as its main advantage, for instance, broad integration of databases such as Graphite, Prometheus, Influxdb, ElasticSearch, MySQL, PostgreSQL, etc. The tool is useful for time series analysis, so it is widely used for monitoring variables or for the development of Key Performance Indicators (KPIs). Grafana can be executed “on-premise ”, which means that it can be deployed within the institution for organizations that do not want their data to be transmitted to a vendor cloud for security or other reasons. The organization has complete control of the infrastructure; the data remain on their private network, and no one but their team has access to the information. Besides, Grafana offers a range of visualization options such as geographic maps, heat maps, histograms, and the full range of charts and graphs that a business typically requires to study the data.

In Grafana, there are defined roles that determine the resources you are allowed to access. There are three types of roles: admin, editor, and viewer.

- Admin: can do everything scoped by the organization, for example add, edit, delete data sources, teams, folders containing dashboards, etc.
- Editor: can view, add, and edit dashboards, panels, and alert rules in dashboards to which they have access; cannot add, edit, or delete data sources; these data can be disabled for specific folders and dashboards.
- Viewer: can visualize any dashboard and data if they have access; cannot manage other organizations, users, and teams.

For the network layer, we only use two of three profiles, admin performing as the manager profile and viewer as the viewer profile.

The sensed data are stored in a MySQL database. Grafana presents a set of tools for consultation that are useful for data visualization, as well as analysis.

Data visualization can be done by accessing https://usuariografana.grafana.net and signing in with the username: usuariografana@gmail.com and the password: Grafana123. At the start page in the home section (upper Left), a dashboard called “weather station” can be found, and once opened, at the upper right area, in the time configuration option, a time range interval from “19 October 2020 23:38:22” to “19 October 2020 23:55:11” can be introduced and applied; the sensors’ data will be displayed.

## 5. Application to Education Engineering within the Educational Mechatronics Framework Using the MEIoT Weather Station

The OBNiSE architecture allows us to integrate and perform applications in several areas, such as mobility, health, smart cities, technology, and education. In particular, this proposal presents the application of engineering education that aims to develop skills and abilities required by I4.0 and promote active learning using resources, existing academic spaces, practical activities, and mechatronic prototypes based on an innovative educational methodology [14]. These courses can be synchronous or asynchronous, and it is an alternative to improve and reduce the gap between the current knowledge at schools and the new industry requirements. The instructional design based on the EMCF is presented below.

### Instructional Design

The instructional design is focused on the subject of dynamical systems, and the mathematical model of Newton’s law of cooling is represented by a first-order differential equation. The EMCF involves three perspective entities: dynamics (process) + Internet of Things (application) + MEIoT Station (artifact). The mechatronic concept is presented and defined in [30] as:

“A mathematical model of a dynamical system is defined as a set of equations that represent the dynamics of the system accurately, or at least fairly well”.

Now, the pedagogical activities for the three levels with the selected perspective, which are the concrete learning level, graphic learning level, and abstract learning level, are defined as follows.

**Figure 8:**
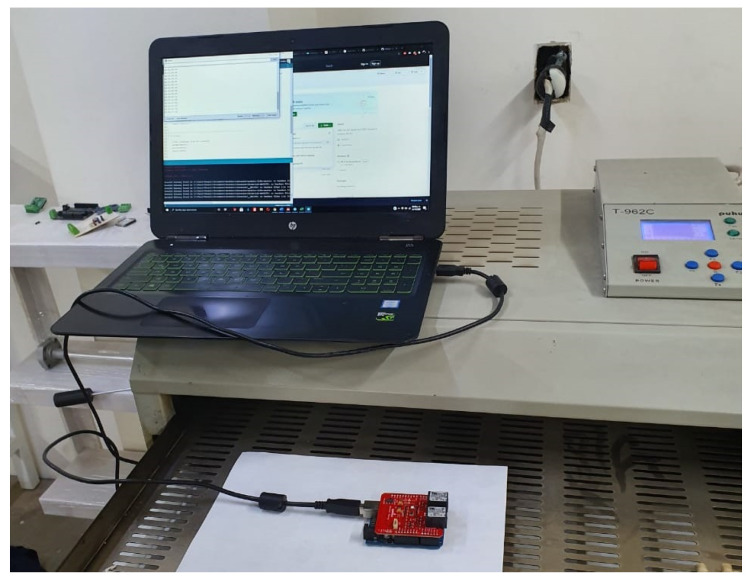
Concrete level: placing the temperature sensor above the infrared heater.

**Figure 9:**
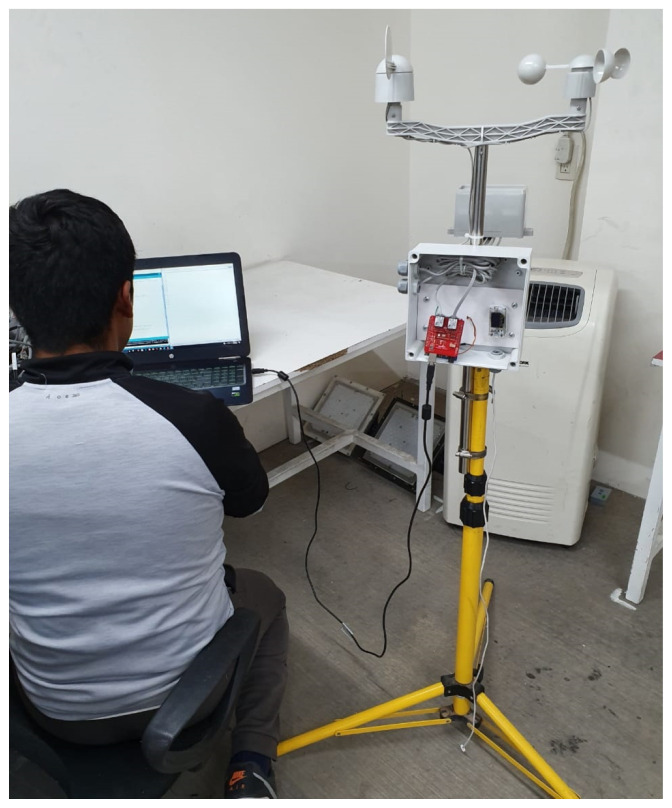
Concrete level: MEIoT Weather Station.

**Figure 10:**
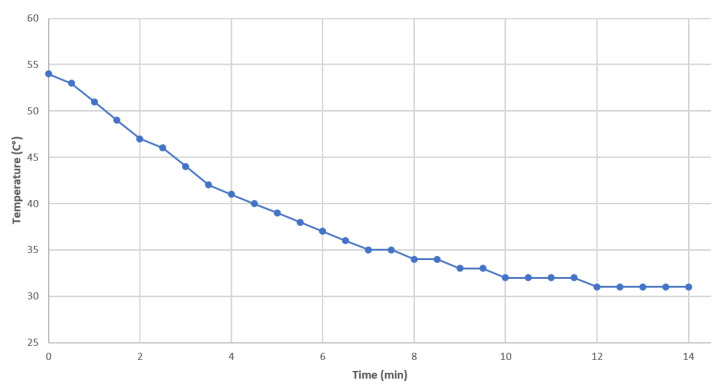
Instructional design: graphic level showing the drawing of the plot considering time vs. measured temperature.

- Concrete Learning Level (CLL): At this level, activities aimed at perceptual-motor characteristics should be designed using the MEIoT Weather Station (see Figure 8 and Figure 9). The activities to perform at this level are described as follows.
  1. Turn on the MEIoT Weather Station.
  2. Measure the environment temperature and record it.
  3. Place the temperature sensor above the T962C infrared heater reflow oven.
  4. Turn on the infrared heater reflow oven to increase the temperature value above the environment temperature, e.g., 54 °C
  5. Turn off the infrared heater reflow oven.
  6. Write the temperature values in a two-column table, with time and measured temperature, every 30 s using the Arduino terminal interface.
  The data collection registered by the participant is shown in (Table 1).
- Graphic Learning Level (GLL): At this level, activities aimed at the graphic (symbolic) representation of mechatronic concepts should be designed, taking as a reference the concepts learned previously at the concrete learning level. The learning will gradually make the transition from the concrete to the abstract level. The graphical level is carried out using the data collected by the participant (see Table 1). Tasks related to this level are described below.
  1. On a white paper sheet, the participant plots each temperature value with points.
  2. Starting from the first point (initial condition), join all points using smooth lines.
    Remark: Here, the participant draw the pencil to meet all the points dynamically. Figure 10 depicts the resulting plot.

**Figure 11:**
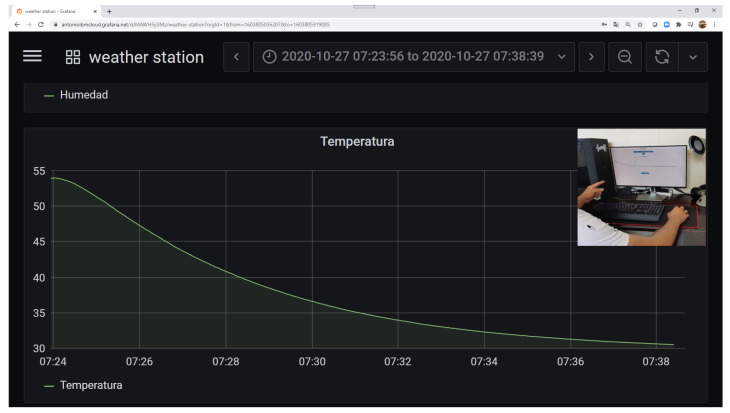
Instructional design: graphic level showing the data of the weather station in real time with the open-source platform.

Now, for the graphical level to be more significant, the online open-source platform Grafana is used to display the collected temperature data. The participant can visualize the cooling dynamics of the sensor and how the temperature value is decreasing as time passes until reaching the environment temperature (see Figure 11).

- Abstract Learning Level (ALL): At this level, activities should be designed to gradually transition from symbolic concepts to abstract representation that include mathematical equations. The differential equations describing Newton’s law of cooling are defined by Equation (1).
  (1) $$\frac{dT}{dt}=k\left(T-T_{m}\right)$$

Consider the definition of the following variables:

T→temperature

t→time

$T_{m}\to \;\mathrm{environmental}\;\mathrm{temperature}$

k→proportionalityquotient

Applying the resolution method of independent variables, the procedure presented in Equation (2) is obtained.

(2) $$\int \frac{dT}{T-T_{m}}=\int kdt$$

$$\begin{array}{c} e(ln\left(T-T_{m}\right)=Kt+C_{i})\\ T-T_{m}=e^{kt+C_{i}} \end{array}$$

T−Tm=ekteCiC

$$\begin{array}{c} \mathrm{Solution}:\;T\left(t\right)=Ce^{kt}+T_{m} \end{array}$$

Now, we have two unknown constants in the solution of the differential equations: *C* and *k*. In order to solve this, we need at least two initial conditions. The recommended one are when the time equals zero and in the inflection point of the graph, which in this case is when the time equals 5 min. The data gathered are:

$T_{m}=31$ °C

T(0)=54 °C

T(5)=39 °C

Substitute T(0)=54 °C in the solution of Equation (2). The result of this substitution is shown in Equation (3).

(3) $$T\left(0\right)=Ce^{k(0)}+31$$

54=Ce0+31

$$\begin{array}{c} C=54-31=23\\ \mathrm{Solution}:\;T\left(t\right)=23e^{kt}+31 \end{array}$$

Now, substitute the initial condition T(5)=39 °C in Equation (3). The procedure presented in Equation (4) is obtained.

(4) $$T\left(t\right)=23e^{kt}+31$$

$$\begin{array}{c} T\left(5\right)=23e^{k5}+31\\ 39=23e^{5k}+31\\ 39-31=23e^{5k}\\ ln(\frac{8}{23}=e^{5k})\\ ln\left(\frac{8}{23}\right)=5k\to k=\frac{ln(\frac{8}{23})}{5}=-0.21121 \end{array}$$

Finally, the solution of the differential equation is presented in Equation (5). This is an expression for the dependent variable *T* in terms of the independent one *t*, which satisfies the relation:

(5) $$T\left(t\right)=23e^{-0.21121t}+31$$

**Figure 12:**
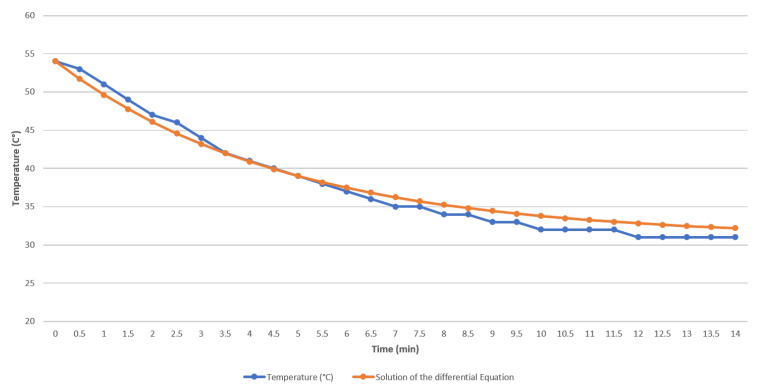
Measured temperature and temperature obtained with the solution of the differential equation.

Table 2 shows the data of the measured temperature and the temperature obtained with the solution of the differential equation against time. It is worthwhile to note that the values are approximate (see Figure 12).

**Figure 13:**
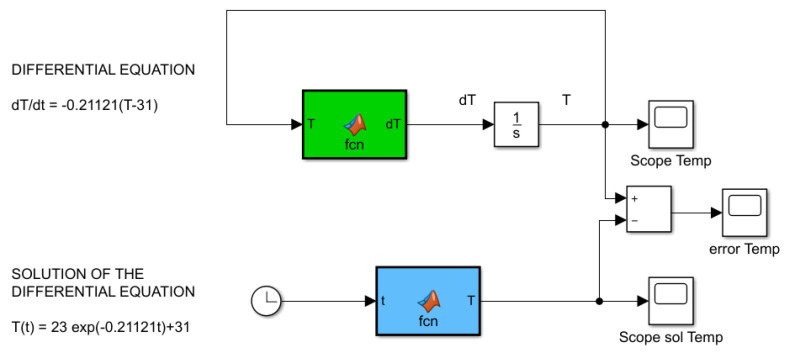
Instructional design: abstract level.

Now, we can implement in Simulink both the differential equation in Equation (1) and the solution of this differential equation in Equation (5). The block diagram for the simulation of the mathematical model of Newton’s cooling law is depicted in Figure 13.

**Figure 14:**
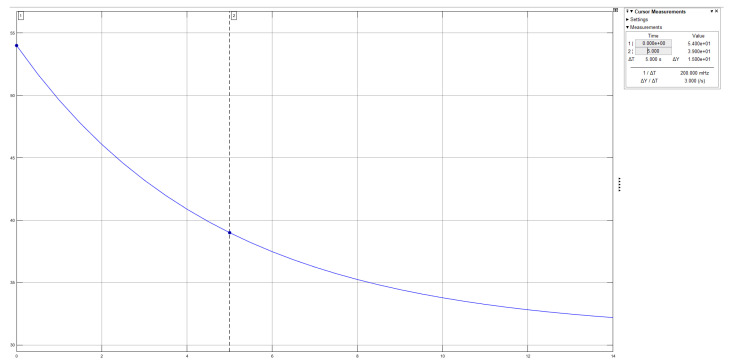
Simulink plot of the solution of the differential equation.

The plotted graph in Simulink is shown in Figure 14.

**Figure 15:**
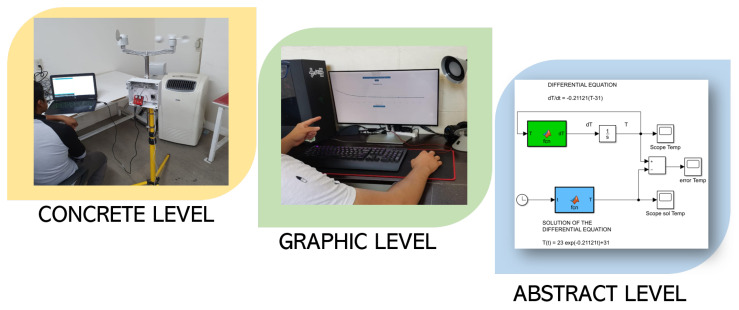
Instructional design.

As a summary, Figure 15 shows the complete instructional design with the three main levels applying the EMCF.

It is worthwhile mentioning that the concrete level presenting the process to build the MEIoT Weather Station could be followed by any person with an engineering background and who understands the required hardware. In the case of the software, as it is open-source, implying ease of use and understanding for users; only simple configurations to the model are required. Finally, for the abstract level, the proposal gives the procedure of describing, solving, and implementing a simulation of a differential equation and Newton’s cooling law, which can be easily extended for more applications.

## 6. Discussion

This novel MEIoT Weather Station was designed and developed as a compact device that allows weather variables’ monitoring in a defined period every second. This station can be easily adapted to any environment without the need for an expensive infrastructure installation, unlike the current solutions. The three main elements and levels working together (concrete, graphic, and abstract) in the proposed architecture and educational mechatronics knowledge are considered to be understood by any student to reduce the educational gap between the current system with the needs of I4.0. The OBNiSE architecture could result in a profound change in educational paradigms, and it could encompass many more areas by generating new artifacts from different applications and process.

It should be noted that the MEIoT Weather Station can be made using sensors available on the market. The open-access platform allows this solution’s realization to be achievable, and it can be implemented anywhere where connectivity to the Internet is available; it also has an Internet of Things basis, which makes it attractive to be developed. Furthermore, the architecture allows the integration of heterogeneous devices to be adaptable and configurable at any level of the architecture, and it is easy to understand.

## 7. Conclusions and Future Work

This work presents the development of an IoT device under an educational framework that allows the development of educational tools so that students can acquire and reaffirm knowledge while becoming familiar with the tools available in I4.0.

The MEIoT Weather Station is just the first of many possible IoT devices that can be used in the OBNiSE architecture within the EMCF for applications in engineering education. The example developed in the reinforcement of the learning of dynamic systems and differential equations’ modeling was obtained using only one of eight climatic variables that the MEIoT Weather Station can monitor.

This article also clearly exposes the possibility of developing ad hoc systems for the application at the same time as the educational framework is included, which allows visualizing and interacting with elements or concepts in a three-level process: concrete, graphic, and abstract, something that was not found in the cited literature.

Finally, the development of a device such as the MEIoT Weather Station and its use as a pedagogical tool under the conceptual framework of educational mechatronics represents an innovative alternative to develop knowledge and skills in the area of the Internet of Things (IoT) in I4.0. Through the implementation and use of a device with characteristics that are easy to understand and follow, the MEIoT Weather Station contributes significantly to the daily academic work, since in this way, it is possible for different universities and professional training centers to use it simultaneously. In such a way, many students can obtain IoT training by applying it from initial practices with gradually increasing difficulty levels.

As future work, tests could be carried out with the number of users and devices connected to the platform to observe its performance. Additionally, the connection with the IoT platform can be improved so that the data are stored internally. Another proposal is the implementation of this conceptual model of education in several universities to make improvements to the device or the architecture and measure the impact of this system on students and higher education institutions.

## Figures and Tables

**Table 1:** Data collection of the measured temperature by the participant.

Time (Min)	Temperature (°C)
0	54
0.5	53
1	51
1.5	49
2	47
2.5	46
3	44
3.5	42
4	41
4.5	40
5	39
5.5	38
6	37
6.5	36
7	35
7.5	35
8	34
8.5	34
9	33
9.5	33
10	32
10.5	32
11	32
11.5	32
12	31
12.5	31
13	31
13.5	31
14	31

**Table 2:** Data collection of the measured temperature by the participant.

Time (Min)	Temperature (°C)	Solution of the Differential Equation (°C)
0	54	54.0000
0.5	53	51.6949
1	51	49.6209
1.5	49	47.7547
2	47	46.0755
2.5	46	44.5647
3	44	43.2052
3.5	42	41.9820
4	41	40.8814
4.5	40	39.8911
5	39	39.0000
5.5	38	38.1983
6	37	37.4768
6.5	36	36.8277
7	35	36.2437
7.5	35	35.7182
8	34	35.2453
8.5	34	34.8198
9	33	34.4370
9.5	33	34.0926
10	32	33.7826
10.5	32	33.5037
11	32	33.2528
11.5	32	33.0270
12	31	32.8239
12.5	31	32.6411
13	31	32.4766
13.5	31	32.3286
14	31	32.1955

## Data Availability

The data presented in this study are available in Table 2.

## Abbreviations

The following abbreviations are used in this manuscript.

ADC	Analog to Digital Converter
AGV	Automated Guided Vehicle
ALL	Abstract Learning Level
API	Application Program Interface
CAD	Computer-Aided Design
CMOS	Complementary Metal Oxide Semiconductor
CIIDETEC-UVM	Centro de Investigación, Innovación y Desarrollo Tecnológico, UVM
CLL	Concrete Learning Level
CPU	Central Processing Unit
CSV	Comma-Separated Values
EMCF	Educational Mechatronics Conceptual Framework
GLL	Graphic Learning Level
GUI	Graphic User Interface
IC	Integrated Circuit
ICT	Information and communication Technology
I4.0	Industry 4.0
IoT	Internet of Things
I2C	Inter-Integrated Circuit
KPI	Key Performance Indicators
MEIoT	Educational Mechatronic Internet of Things
MEMS	Microelectromechanical Systems
MCU	Microcontroller Unit
MQTT	Message Queuing Telemetry Transport
OBNiSE	Observatorio Nacional Digital de Smart nvironments
RoHS	Restriction of Hazardous Substances
SCARA	Selective Compliant Articulated Robot Arm
SMD	Surface Mount Device
TLS	Transport Layer Security
UVM	Universidad del Valle de México
VNC	Virtual Network Computing
